# Embryonic Mouse Cardiodynamic OCT Imaging

**DOI:** 10.3390/jcdd7040042

**Published:** 2020-10-04

**Authors:** Andrew L. Lopez, Shang Wang, Irina V. Larina

**Affiliations:** 1 Department of Molecular Physiology and Biophysics, Baylor College of Medicine, One Baylor Plaza, Houston, TX 77030, USA; all1@bcm.edu; 2 Department of Biomedical Engineering, Stevens Institute of Technology, Castle Point on Hudson, Hoboken, NJ 07030, USA; swang148@stevens.edu

**Keywords:** mouse, cardiogenesis, in vivo imaging, optical coherence tomography, embryo culture, cardiodynamics

## Abstract

The embryonic heart is an active and developing organ. Genetic studies in mouse models have generated great insight into normal heart development and congenital heart defects, and suggest mechanical forces such as heart contraction and blood flow to be implicated in cardiogenesis and disease. To explore this relationship and investigate the interplay between biomechanical forces and cardiac development, live dynamic cardiac imaging is essential. Cardiodynamic imaging with optical coherence tomography (OCT) is proving to be a unique approach to functional analysis of the embryonic mouse heart. Its compatibility with live culture systems, reagent-free contrast, cellular level resolution, and millimeter scale imaging depth make it capable of imaging the heart volumetrically and providing spatially resolved information on heart wall dynamics and blood flow. Here, we review the progress made in mouse embryonic cardiodynamic imaging with OCT, highlighting leaps in technology to overcome limitations in resolution and acquisition speed. We describe state-of-the-art functional OCT methods such as Doppler OCT and OCT angiography for blood flow imaging and quantification in the beating heart. As OCT is a continuously developing technology, we provide insight into the future developments of this area, toward the investigation of normal cardiogenesis and congenital heart defects.

## 1. Introduction

The mouse is an invaluable tool to study cardiogenesis. The development of the embryonic mouse heart parallels human heart development in gene expression, structure, and function, providing investigators a biological platform to delineate human heart development and root causes of cardiac disease. The mouse’s genome is highly versatile to genetic manipulation in comparison to other mammalian models and is amplified by a supportive scientific industry that maintains and provides mouse models and gene editing services. By these means, investigators have identified key genes: that determine cardiac cell fate [1,2,3], regulate size and proliferation [4,5], septate and divide chambers [6], and several more including extracellular matrix formation [7,8] and electrical signaling [9,10]. Further, the recapitulation in mice of chromosome deletions and gene mutations identified in congenital heart disease (CHD) patients that generate similar phenotypes is our most robust set of data that affirms the genetic source of CHD in human patients [11]. As a result, we have to come to understand the developing heart as a transitional organ that executes a highly orchestrated patterning of gene programs to guide a primordial bed of cells in the mesoderm to form a functional chamber and valve pump. To better appreciate the dynamic cell biology that takes place during heart development, we have to experiment with the heart in its natural state, as a growing mechanical pump, forcing fluid through the heart tube and vasculature. To this end, over the last 20 years, studies in biophotonics has implemented cardiodynamic imaging through optical imaging modalities and live culture protocols to visualize and quantify structure and function of the beating heart through development [12,13,14,15,16,17]. The combinatorial approach of cardiodynamic imaging and utilization of genetic mouse models holds promise to inform us on the relationship between the heart’s function, its cell biology, and the origins of congenital heart disease.

In the mouse, heart development starts on embryonic day (E)7.5. Hallmark events—the transition from cardiac crescent to linear tube, first contractions and rightward looping, chamber formation and trabeculation—occur in the first two days when the heart is only hundreds of microns in size, while the embryo resides in utero embedded in decidua (a thick layer of modified mucous membrane which lines the uterus) and covered by layers of maternal tissue. This has created a challenge for imaging cardiodynamics in the mouse embryo. High-resolution magnetic resonance imaging (MRI) was utilized for longitudinal in utero imaging of mouse embryos from E10.5 to E14.5, showing three-dimensional (3D) anatomical structures as well as the developing vasculature [18]. Although an isotropic 100 µm spatial resolution can be achieved with MRI that reveals cardiac morphology, the dynamics of the heart is missing from such analyses due to the relatively low temporal resolution [19]. High-frequency ultrasound allows in utero cardiodynamic imaging of mouse embryos staged between E8.5 and E13.5, revealing atrial and ventricular contractions as well as longitudinal changes in the cardiac morphology [20]. Quantitative characterization of blood flow dynamics in mouse embryos was also performed over different gestational stages [21]. However, the spatial resolution is limited to 30 µm in the axial direction and 90 µm in the transverse direction, which is not sufficient to resolve detailed structures of the embryonic heart, such as the endocardium, and does not allow for reliable cardiac measurements for the small embryonic heart at stages E8.5 to E9.5 [20]. Optical imaging modalities have a promise to overcome such limitations due to their high spatiotemporal resolution [12]. Due to a millimeter or sub-millimeter level imaging depth, optical techniques cannot provide noninvasive in utero imaging for early-stage mouse embryos, but advancements in live mouse embryo culture paired with optical modalities have enabled unprecedented visualizations and analyses of mouse embryonic cardiovascular dynamics [17,22,23]. A comparison of different imaging modalities for embryonic imaging was recently reviewed by Wang et al. [22].

Optical coherence tomography (OCT), a low-coherence interferometric technique, has an ideal imaging scale for the mouse embryo [24]. The spatial micron resolution and millimeter-level imaging depth place its imaging capabilities between confocal microscopy and high-frequency ultrasound [13]. The early work by Boppart et al. explored the use of OCT for morphological and anatomical imaging of *Xenopus* and zebrafish embryos, setting the stage for OCT in developmental biology [25,26,27]. Rapid advancements in the OCT field since the 1990s placed OCT in a unique position for a number of critical applications in embryonic imaging. Most importantly, the emergence of Fourier-domain OCT brought higher imaging speed and much improved sensitivity which allowed for capturing the dynamics of the embryonic mouse heart at early stages in its entirety [28,29]. The development of various functional OCT methods, such as Doppler OCT and OCT angiography, enabled additional imaging contrast to study the hemodynamics and vascular system of embryos without labeling [30,31,32], which established OCT as a powerful imaging tool for phenotyping the structure, morphology and function of the developing heart and vasculature in the mouse model.

In this review, we highlight advances in OCT mouse embryonic cardiodynamic imaging over the last 12 years. We describe live imaging approaches and advances in image processing. We highlight advances in OCT imaging technology which represent leaps in performance to overcome limitations in resolution and acquisition speed for cardiodynamic imaging. Finally, we bring our perspective on future directions and questions to consider toward advancement of biophotonic cardiodynamic study in embryonic mouse models.

## 2. Mouse Embryonic Imaging in Static Culture

In early attempts to image mouse embryonic cardiodynamics with OCT, Jenkins et al. performed OCT imaging of extracted E13.5 hearts, which were paced externally for gated acquisition and dynamic volumetric reconstruction [33]. At about the same time, Luo et al. performed OCT imaging of the beating heart in freshly dissected E10.5 mouse embryos [34], though the heartrate of the embryo was significantly reduced. The potential for OCT technology in structural and functional analysis of mouse embryonic hearts uncovered by these early studies became greatly expanded by integration of OCT technology with robust static mouse embryo culture protocol [31], which is critical for maintenance of embryos in semi-physiological state and has a long history of development and optimization.

The first mouse embryo culture experiments were conducted by John Hammond in 1949, the first director of the new Animal Reproduction Unit of the Animal Research Station in Cambridge, UK [35]. This technology made possible the first observations of embryo cleavage and blastocyst formation in mouse embryos. In the following years, investigators established more robust culture protocols that involved careful control of temperature, pH, osmolarity, and air quality. Even with these advances, challenges such as “cell block,” where embryos would undergo cellular arrest if harvested at the zygote stage, were still a major challenge. As the Human Genome Project was starting in the 1990s and more interest was being placed on human embryo development, independent researchers and researchers part of the National Cooperative Program on Non-Human In Vitro Fertilization and Preimplantation Development at the National Institutes of Health, focused on standardizing embryo culture mediums that solved the issue of cell block and provided the foundation for many of the protocols and recipes used to this day [36].

**Figure 1:**
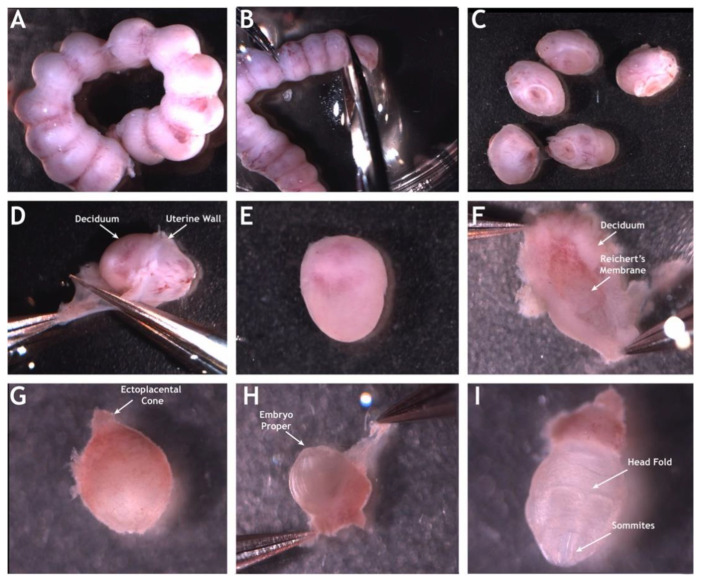
Mouse E8.5 embryo dissection for OCT imaging in static culture. The uterine horn is dissected from the female and separated into individual embryos (**A**–**C**). The uterine wall is peeled away exposing the embryo encapsulated in deciduum (**D**,**E**). The deciduum is peeled away exposing the embryo proper within Reichert’s membrane (**F**–**H**). Panel (**I**) shows the embryo proper attached to its ectoplacental cone. The embryo proper with its intact yolk sac exhibits the open head stage indicating the embryo is E8.5 (**I**). Reproduced with permission from [17].

Now embryo culture systems can be built with more versatility, for a broader range of applications including live imaging [37,38,39]. Particularly, the combination of the static embryo culture with the OCT technology provides us with one of the highest resolution modalities to image heart functionality in developing mouse embryos [40,41]. Modern embryonic mouse static culture protocols [42] involve careful micro-dissections on a temperature controlled microscope station (Figure 1). After dissection, embryos recover in an environmentally controlled incubator and are then OCT imaged while in culture media under environmental control. For that, the sample arm of the OCT apparatus is housed within a commercial incubator maintained at 37 °C and 5% CO_2_ to sustain the embryo for multiple hours. Prolonged OCT imaging in static embryo culture has been demonstrated, where rat serum was added to the culture medium to better support the embryo growth and a thin piece of Teflon film and mineral oil were placed on top of the medium to prevent evaporation [40]. This setup allowed for imaging of embryonic development for more than 16 h, providing visualization of the cranial neural tube closure. While not the perfect physiological environment, this approach allows imaging of embryonic development with minimal perturbations.

## 3. Approaches to Volumetric Cardiodynamic Imaging

Within the first 24 h after the beginning of contractions, the whole depth of the heart can be imaged with OCT in live mouse embryo culture to allow high-resolution visualization of cardiac features; however, a high volume acquisition rate of ~100 Hz is required to characterize cardiodynamics at these stages. Because OCT is a scanning technology, traditional OCT imaging is too slow to capture the dynamics of heartbeat volumetrically. To solve this limitation, a number of synchronization algorithms have been developed for embryonic cardiodynamics. These methods take advantage of heartbeat periodicity and assume no structural or functional changes of the cardiovascular system during acquisition. Some approaches, which have been demonstrated for OCT imaging of *Xenopus*, chicken and excised mouse hearts [33,43], rely on a separate gating signal to register time sequences acquired at different locations of the heart according to the heartbeat phase. These methods require additional hardware to collect the gating signal and position two imaging systems around the embryo, which is particularly challenging for very small and environmentally sensitive cultured mouse embryos.

**Figure 2:**
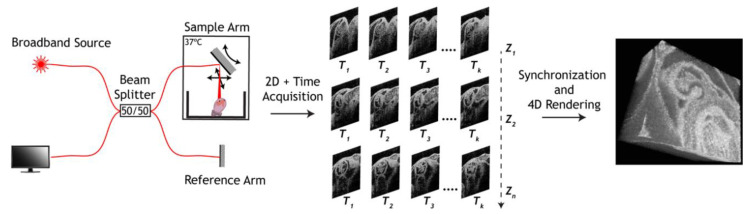
4-D OCT cardiodynamic embryonic imaging in parallel slice geometry. Reproduced with permission from [41].

The most popular approach used for mouse embryonic cardiodynamic reconstruction was originally developed by Liebling et al. for confocal microscopy [44,45], then adapted for OCT [29,31]. For this method, two-dimensional time-lapses are acquired sequentially in parallel slice geometry with a small step through the whole heart, each time-lapse covering at least two full heartbeat cycles. Retrospectively, all the time-lapses are sequentially synchronized to the same phase of the heart beat cycle based on the imaging data itself, leaving one complete heartbeat cycle for each position. Volumetric rendering of these synchronized time-lapses provides a 4-D (3-D + Time) visualization of embryonic cardiodynamics at the volume rate identical to the frame rate of the time-lapses (Figure 2).

A recent improvement to this approach involves continuous acquisition of a single dense OCT volume instead of multiple time-lapses at discrete locations [46]. The volume consists of ~15,000–30,000 frames, acquired at 100 fps, and covers an entire heart. The data set is split into subsets corresponding to individual heart beats, and each subset is assigned to a spatial location. The frames in each subset are doubled to produce two complete (identical) uninterrupted heart beats required for the synchronization algorithm. These sequences are synchronized to yield one heartbeat cycle for each location, therefore, eliminating all duplicated frames. This acquisition approach is more flexible because it does not require one to measure the heartrate before the imaging to ensure at least for two uninterrupted heartbeats for each position. It is also much faster because it utilizes all of the acquired frames in the final 4-D reconstruction, in contrast to disposing more than half of the acquired frames, as was done originally.

**Figure 3:**
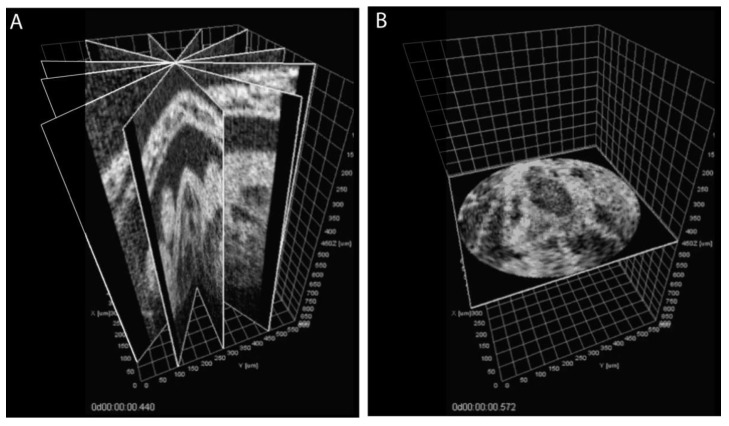
Sequential Turning Acquisition and Reconstruction (STAR) method for embryonic cardiodynamic analysis. (**A**) Representative cross-sections through the synchronized reconstruction demonstrating the positions of the imaging planes (6 out of 60 angles). (**B**) XY cross-section of STAR data after polar-to-Cartesian interpolation. Reproduced with permission from [47].

The described method of reconstruction based on parallel-slice geometry works efficiently and has been successfully used in multiple studies, however, since the sequences are synchronized recursively, it can be susceptible to error propagation. To avoid the propagation errors, an approach was proposed, called the Sequential Turning Acquisition and Reconstruction (STAR) method [47]. This method is based on time-lapses acquired sequentially in radial geometry by rotating the imaging plane around the central line, in star-like geometry (Figure 3). The central intersecting line of all the time-lapses was positioned over the beating heart to provide intrinsic reference for synchronization for all the sequences without propagation error. This method was successfully demonstrated for 4-D cardiodynamic reconstruction, but did not gain popularity primarily because not all OCT imaging systems allow for easy angular adjustment of the imaging plane. In addition, because the data were acquired in star-like geometry, it resulted in non-isotropic resolution for the 4- D reconstruction with resolution degrading away from the central rotation axis.

Another approach without the error propagation but also providing an isotropic resolution, utilized two orthogonal sets of parallel OCT slice-sequences [48] for cross-synchronization and 4-D reconstruction of the embryonic heartbeat. The spatial intersections between all the time-lapses acquired in perpendicular directions were used to spatio-temporally register the slice-sequences. Because each slice-sequence intersected all slice-sequences in a perpendicular data set, it contained multiple references for synchronization and self-validation at each location. This method required twice longer acquisition time than traditional methods relying on a single data set in parallel-slice geometry, but provided more accurate registration, as was demonstrated through phantom simulations and successful reconstruction of cardiodynamics in cultured mouse and rat embryos [48].

**Figure 4:**
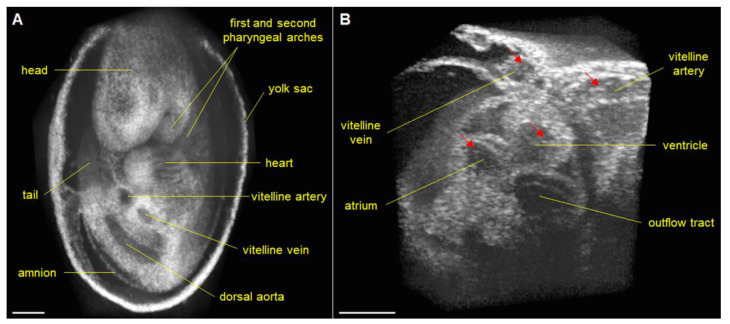
Direct 4-D embryonic imaging with 1.5 MHz OCT. (**A**) Large-field 3D OCT image of E9.5 mouse embryo obtained with slow galvanometer mirror scanning. (**B**) 4-D cardiodynamics of E9.5 mouse embryo obtained from direct time-lapse 3D imaging with a volume acquisition rate of ~43 Hz. Arrows indicate the blood cells. Scale bars are 300 μm. Reproduced with permission from [49].

Technological advances in OCT system development provide new opportunities for embryonic cardiodynamic imaging. New developments in MHz range swept-source laser technology allowed the building of ultra-fast OCT imaging systems capable of direct volumetric cardiac imaging. Wang et al. developed a 1.5 MHz swept-source OCT system and applied it for direct 4-D imaging of E9.5 mouse embryonic cardiodynamics and hemodynamics in static culture at ~43 Hz volume rate (Figure 4) [49]. This advancement allowed the total imaging time to be reduced to less than a second, avoiding potential errors associated with alignment and synchronization, while providing sufficient level of detail to reconstruct embryonic cardiodynamics and characterize spatially resolved heart wall and blood flow dynamics.

With a limited OCT scanning rate, the quality of visualization is achieved through a tradeoff between the frame rate, scan size, pixel density, and number of averages. Improving any of these parameters always comes at the expense of the others. Toward this limitation, an approach has been developed to improve spatial and temporal resolution and the field-of-view through post-acquisition mosaicking of cardiodynamic time-lapses acquired with spatial overlaps [50]. This approach extended traditional static tiling or mosaicking to dynamic time-lapses through combined spatial and temporal registration algorithms, providing an opportunity for significantly increasing the imaged area without reduction in the frame rate or spatial resolution. Noise reduction algorithms for OCT imaging of embryonic hearts have also been developed [51]. This method aligns image-sequences from multiple cardiac cycles through an elastic registration algorithm to account for slight variations between the heartbeats, and averages them into a single cycle. This noise reduction algorithm has been applied to OCT images of the beating heart in cultured mouse embryos, significantly improving the signal-to-noise ratio and revealing structural features which were not visible in the original images. Because this method works by averaging corresponding frames from multiple cycles, it preserves the spatial and temporal resolution, but requires longer total imaging time, which is an important factor to consider for 4-D cardiodynamic acquisition.

## 4. Cardiac Phenotyping of Genetic Mouse Models

OCT imaging is a powerful approach to investigate structural features in mouse hearts and perform structural and functional characterization of mutant phenotypes. Early on, Jenkins et al. applied OCT to imaging extracted hearts from HEXIM1 (hexamethylene-bis-acetamide-inducible protein 1) mutant mouse embryos [14]. The analysis of 3-D OCT cardiac images revealed structural differences between the HEXIM1 mutants and the littermate controls at E12.5 and E13.5, uncovering the great potential for this technology to visualize and quantify structural cardiac defects in mouse mutants toward understanding congenital heart defects in humans [14].

**Figure 5:**
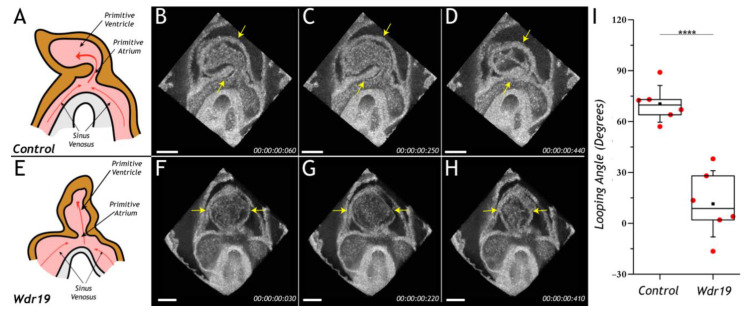
Cardiodynamic analysis in E8.5 Wdr19 embryos. Illustrations represent cross-sections through 4-D OCT volumes at different phases of the heartbeat in control (**A**–**D**) and Wdr19 mutant (**E**–**H**). (**I**) Box plot characterization of cardiac looping in Wdr19 mutants. Red dots show the measured values, solid squares represent the mean, and whiskers correspond to the standard deviation. The number of analyzed embryos N = 6 for both control and mutant groups. **** *p* < 0.0001 from a two- sample two-tailed Student’s *t* test. The scale bars correspond to 200 μm. Reproduced with permission from [41].

Lopez et al. implemented 4-D OCT imaging for structural and functional phenotyping of mouse Wdr19 mutant embryos in culture [41]. Wdr19 is a ciliary protein linked to certain ciliopathies in humans. Wdr19 homozygous mice are embryonic lethal at ~E10.5 with defects in neural tube closure and brain development. OCT imaging study uncovered previously undetected cardiac looping phenotype in addition to previously known neural tube closure defect in Wdr19 mutant embryos, which was apparent at the E8.5 stage and became more severe at the E9.5 stage. 4-D cardiodynamic analysis in E8.5 Wdr19 mutant embryos revealed strong cardiac contractions, blood circulation, and a heartrate similar to the controls (Figure 5). However, the looping angel in the mutant hearts was significantly lower in comparison to the controls, suggesting a role for Wdr19 in regulation of left–right asymmetry during heart development. This study is the first application of 4-D OCT for dynamic cardiac analysis in mouse mutants demonstrating great potential for this technology for functional phenotyping of genetically engineered mouse lines with cardiac failure or embryo demise.

Cardiac morphogenesis and functional maturation are highly regulated and inter-dependent, with any structural or functional deviation at the early stages leading to congenital heart defects or cardiac failure. Cardiodynamic 4-D OCT analysis allows us to differentiate whether cardiac failure in embryonic lethal mutants is primarily due to a structural or functional defect. In contrast to the Wdr19 mutant embryos, where the defect was found to be primarily structural, 4-D OCT analysis in Mlc2a mutant embryos revealed an early functional defect [52]. Homozygous Mlc2a mice, a knockout of the atrial isoform of the Myosin light chain 2 gene, are embryonic lethal at E10.5 showing heart edema and failure. 4-D cardiodynamic OCT analyses at the E9.0 stage have shown that the Mlc2a mutant hearts are contracting, but the contractility is significantly reduced in comparison to the controls, bringing new opportunity to study developmental consequences of functional defects in genetic mouse models.

## 5. Hemodynamic Imaging with Doppler OCT

Doppler OCT is a powerful approach for 3D quantitative analysis of movements [53], with its primary application in blood flow imaging. Established in the 1990s with time-domain OCT systems, Doppler OCT was initially achieved by measuring the frequency shift in the low-coherence interference signal, which enabled the measurement of the sample movements in the direction parallel to the OCT beam [54,55,56]. Later, the emergence of phase-resolved methods and Fourier domain OCT in the early 2000s transformed Doppler OCT into a high-speed, high-resolution and high-sensitivity approach [57,58,59], which is now widely used for quantitative blood flow imaging. Over the years, a number of signal/image processing approaches have been developed to extract the flow velocity from the optical phase. The performance parameters have been compared in detail by Liu et al. [60], providing the groundwork for the application of Doppler OCT in embryonic imaging.

The initial use of Doppler OCT for cardiovascular imaging of embryos [58,61,62] clearly showed the feasibility of Doppler OCT to capture flow dynamics inside the embryonic heart and blood vessels, and established it as resourceful tool to study cardiovascular development. As the technology matured, studies with Doppler OCT rapidly emerged in a number of animal models [31,43,63,64,65], revealing exciting capabilities that could not be achieved with other imaging approaches. Notably, these have enabled biomechanical analysis of the outflow tract [66], 4-D mapping of cardiac wall shear stress [67], and studying dynamic responses of cardiac flows to physical and chemical interventions modeling congenital heart defects [68,69]. These opened new opportunities to investigate and understand hemodynamics as an important mechanical cue in cardiogenesis.

**Figure 6:**
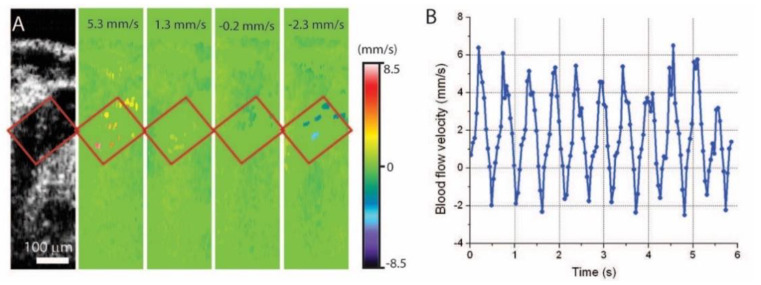
Doppler OCT velocity measurements from individual blood cells in E8.5 mouse embryo. (**A**) Structural and corresponding color coded Doppler velocity images acquired at different phases of the heartbeat cycle. Green corresponds to zero velocity. Individual blood cells are distinguishable in the dorsal aorta. (**B**) Average blood flow velocity as a function of time in the corresponding area of the dorsal aorta. Reproduced with permission from [70].

For the mouse model, Doppler OCT has shown exciting applications for spatiotemporal imaging and quantitative assessment of the hemodynamics in mid-gestation embryos. Initial work provided blood flow profiles of velocity over time in the yolk sac vessel and dorsal aorta that correlated well with observations from confocal microscopy [31]. With the micro-scale resolution of OCT, individual circulating blood cells in the dorsal aorta can be resolved and their velocities can be quantified (Figure 6) [70]. This suggested the high resolvability and sensitivity of Doppler OCT for hemodynamic characterization.

**Figure 7:**
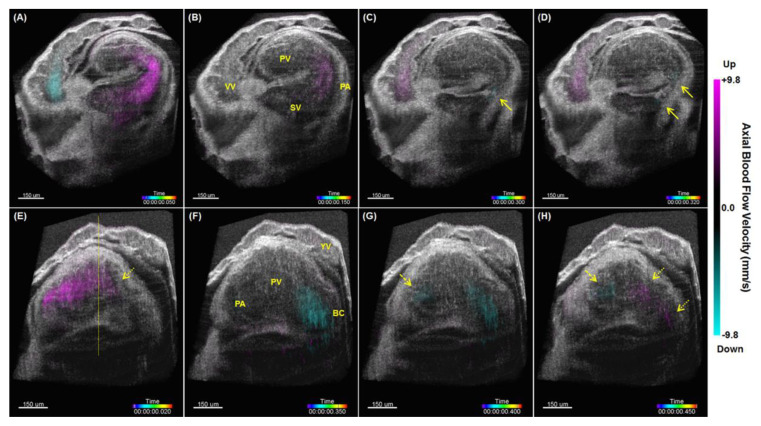
4-D imaging of hemodynamics in the live E9.0 mouse embryonic heart. (**A**–**D**) are cross-sectional views that focus on the sinus venosus, primitive atrium and vitelline vein at different time points of cardiac cycle. (**E**–**H**) are cross-sectional views that focus on the primitive ventricle and bulbus cordis at different time points of cardiac cycle. Solid arrows point at retrograde flows in the primitive atrium. Dashed arrows point at retrograde flows in the atrioventricular region. Dotted arrows point at retrograde flows in the bulbus cordis and the bulboventricular region. YV: yolk sac vessel; VV: vitelline vein; SV: sinus venosus; PA: primitive atrium; PV: primitive ventricle; BC: bulbus cordis. Reproduced with permission from [71].

Through post-processing synchronization, 4-D hemodynamic imaging of the beating heart in the live mouse embryos has been achieved [71]. The synchronization of the Doppler OCT image time-lapses were based on the corresponding structural OCT data as described above. This produced a combined 4-D cardiodynamic and hemodynamic imaging of the embryonic heart. An example is shown in Figure 7. Such 4-D reconstructions enabled a clear visualization and quantitative characterization of the blood flow dynamics throughout the entire heart. In particular, this study revealed strong retrograde flows in the E9.0 mouse embryonic heart, and showed that while, overall net flow is forward, retrograde flows generate higher shear stress at the bulbus cordis region due to luminal constriction, which might be potentially important as mechanotransduction stimulus for proper valve development [71].

Doppler OCT provides a number of unique advantages over fluorescence-based microscopic methods, such as confocal microscopy (CM), which has been widely utilized in studies of cardiovascular development. First, Doppler OCT provides a larger imaging depth that can cover embryonic mouse heart development up to E9.5, which cannot be achieved with CM. Second, Doppler OCT directly generates the flow velocity information, while with CM, a cell tracking approach is required to quantify the flow. Third, Doppler OCT imaging allows for higher imaging speed over CM, which is highly beneficial for cardiodynamic imaging. Finally, Doppler OCT is achieved with endogenous contrast, not requiring any fluorescence labeling. These features have recently enabled detailed pumping assessment of the mouse tubular embryonic heart at E9.25, which revealed interesting pumping dynamics in localized cardiac regions [72]. The unique combination of imaging scale, speed, and contrast of Doppler OCT allows for convenience and improved efficiency in probing cardiac blood flow and enables functional imaging and biomechanical analysis of the developing mouse heart, which otherwise is not possible.

## 6. OCT Angiography Approach for Cardiovascular Imaging

OCT angiography is based on speckle or phase fluctuations in OCT data produced by moving blood cells [73] and allows for hemodynamic imaging without any labeling [74]. For hemodynamic imaging in mouse embryos, the speckle-variance (SV) OCT approach has been primarily used [22], where the variance is calculated for each pixel over multiple OCT structural images over time to map the flow in 3D. SV OCT angiography has a high sensitivity in resolving active blood circulation and enables micro-scale resolution in reconstruction of the microvasculature. There have been multiple technological developments in the area of OCT angiography over recent years, including novel data processing algorithms to improve the signal-to-noise ratio for flow detection [75] as well as the adoption of deep learning models [76]. Such technological advancements open up a number of significant biomedical applications [73,74], including embryonic cardiac functional phenotyping.

**Figure 8:**
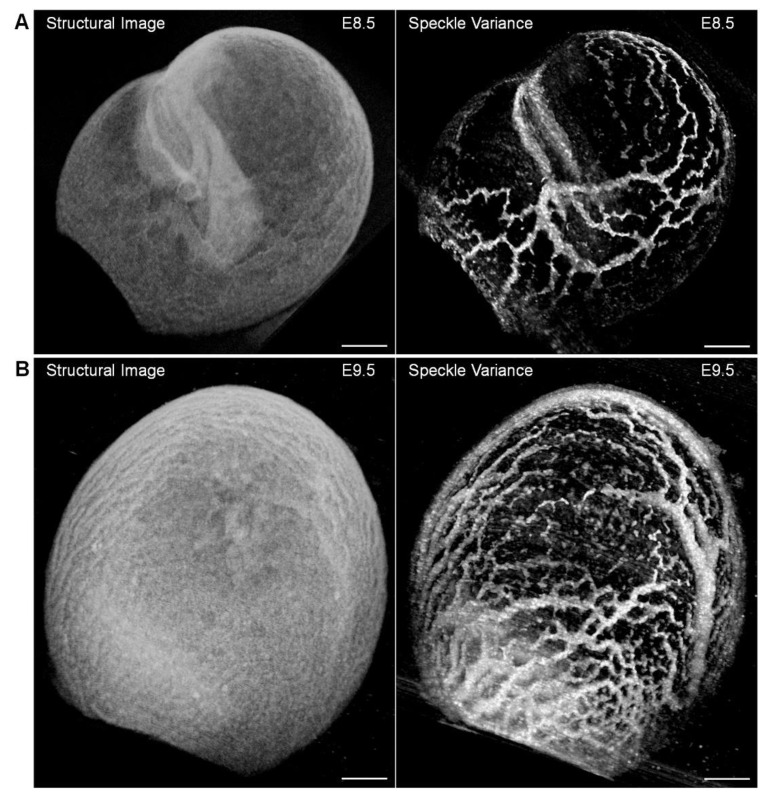
Visualizing the embryonic yolk sac vasculature with structural OCT (**left**) and SV OCT (**right**). Vascular remodeling from (**A**) E8.5 to (**B**) E9.5 is revealed from the speckle-variance (SV) contrast. Scale bars are 300 µm. Reproduced with permission from [77].

Sudheendran et al. [32] and Wang et al. [77] implemented SV OCT angiography to reconstruct the yolk sac vasculature of the mid-gestation mouse embryo. OCT scanning was performed with repeated B-scans at each frame location of the volume, and speckle variance was calculated between the consecutive B-scans at each position. In a comparative analysis, OCT angiography presented advantages over Doppler OCT in resolving the yolk sac blood flow, because Doppler OCT is only sensitive to the axial component of blood flow, while the SV OCT can reconstruct the whole circulatory system regardless of the flow direction [32]. The reconstructions of the yolk sac vasculature have been obtained at different developmental stages, E8.5 and E9.5, clearly showing the vascular remodeling (Figure 8) [77].

An improved OCT angiography reconstruction algorithm has recently been developed by Kulkarni et al. [78] to allow detection of the yolk sac vessel segments that were either partially missed or weakly depicted by the traditional method [78]. This work also presented quantitative evaluations of the reconstructed vascular structures in the mouse embryonic yolk sac [78], providing a useful assay for vascular complexity based on OCT angiography. 3D OCT angiography has also been applied for in utero imaging of E14.5 mouse embryos to study the change of brain vasculature in response to maternal exposures to alcohol [79], cannabinoid [80], and nicotine [81], indicating a live functional imaging platform to evaluate a variety of maternal effects on fetal cardiovascular development.

Using 1.5 MHz OCT, direct volumetric imaging at ~43 Hz was performed by Wang et al. for inter-volume SV calculation from the same phase over six continuous cardiac cycles [49]. The imaging was performed in E9.5 mouse embryo. The inter-volume SV analysis revealed the yolk sac vasculature, vitelline artery, vitelline vein as well as their branches in 4-D at ~43 Hz volume rate [49].

**Figure 9:**
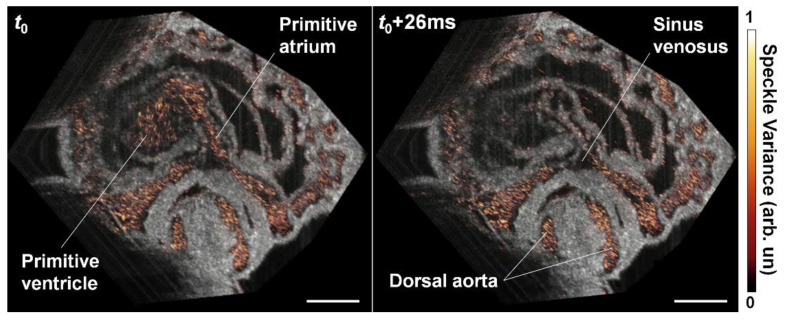
4-D OCT angiography of the beating heart in E8.5 mouse embryo showing the well-separated blood circulations by speckle-variance contrast. Scale bars are 150 µm. Reproduced with permission from [46].

Dynamic 4-D OCT angiography within the beating embryonic heart has recently been achieved by Grishina et al. through synchronization approaches [46]. Instead of comparing the consecutive frames at the same position as is done for traditional speckle variance calculation, the algorithm used frames from multiple cycles but acquired at the same phase of the heart beat cycle. This method relied on the periodicity of heart wall movement and assumed that the circulating blood cells do not return to the same spatial positions over heartbeat cycles [46], which produces a higher variation of OCT speckles in time within the heart and vessels. A single volume of densely-sampled B-scans was acquired over multiple heartbeat cycles for each reconstruction, so that the distance between B-scans at the same phase of adjacent cardiac cycles was set to be smaller than half of the OCT beam size. This ensures that at least two B-scans can be considered at the same spatial location at the same phase of the heartbeat [46]. Such B-scans were used to calculate the speckle variance for each voxel in the heart volume and at each phase of cardiac cycle, providing 4-D OCT angiography of the beating embryonic heart [46]. As shown in Figure 9, SV highlights the blood flow inside the heart and separates well the heart wall from the flow at 100 Hz volume rate.

The major feature of OCT angiography in mouse embryonic imaging is endogenous contrast. Confocal microscopy has been the standard approach for imaging the mouse embryonic yolk sac vasculature, where vessels are labeled by fluorescence protein and the yolk sac is dissected and flattened [37]. In comparison to this, the non-labeling live imaging with a large 3D field of view from OCT angiography is promising to significantly improve the convenience for phenotyping the vasculature network in the mouse embryo. The micro-scale resolution of OCT enables the microvasculature to be resolved even at the early developmental stage. Also, the 4-D imaging capability with a high temporal resolution allows the dynamics of vascular remodeling to be studied. In addition, the functionality of the vessel can be revealed, as only vessels with circulating blood cells provide an imaging contrast. As OCT angiography technologies continues to advance, quantitative angiography, a mapping of the blood flow speed could further boost its significant applications in understanding the mouse embryonic angiogenesis. Since volumetric OCT imaging can be performed in static mouse embryo culture for over 16 h [40], this can be valuable for label-free characterization of the vasculature phenotypes in genetic mouse models. All these together point to an encouraging view that OCT angiography could bring innovative cardiovascular studies of mammalian heart development.

## 7. Perspective on Future Developments

OCT imaging of the mouse embryo is largely enabled by live static embryo culture. Extended culturing capability is a critical aspect in further advancing OCT embryonic imaging [82]. Specifically, longitudinal cardiodynamic imaging over the early developmental stage could be potentially enabled by the improvement of culture conditions and innovative culturing approaches. Prolonged OCT embryonic imaging in static culture has been demonstrated for over 16 h [40]. That same approach can be potentially used for cardiodynamic imaging over development. In comparison with static culture, roller culture provides an optimal ex utero condition for embryonic growth [83]. This culturing technique [84] could be integrated in the imaging protocol, by performing OCT cardiodynamic imaging in static culture at selected time points over the roller culture period, thus achieving longitudinal analysis of the beating embryonic heart for an even longer time of development.

As a powerful imaging modality, OCT has enabled exciting technological advancements that could lead to new opportunities for cardiodynamic analysis. With spectroscopic information, photothermal effect, or pump-probe mechanism, molecular contrast can be achieved with OCT [85], providing the potential for molecular OCT imaging of mouse embryonic hearts. Innovative designs and implementations of OCT probes [86,87] can potentially inspire new strategies of integrating the OCT imaging head with the embryo culturing setup, which might lead to an improved experimental environment and condition for live mouse embryonic imaging. Image processing methods to extract specific features are increasingly demanded for the assessment of rich information provided by OCT, and to this end, deep-learning-enabled algorithms developed for OCT embryonic heart images [88,89] could have a profound impact on increasing the efficiency of analyzing the OCT cardiodynamic data. Microscopic particle image velocimetry (µPIV) is an important technique for the measurement of fluid flows; OCT-µPIV as an advanced high-resolution, depth-resolved PIV method has been demonstrated for dynamic flow imaging of chick embryos [90], which complements Doppler OCT by resolving the flow perpendicular to the OCT imaging beam, promising for robust quantification of hemodynamics in the mouse embryonic cardiovascular system.

Due to the increasingly recognized role of mechanical factors in cardiovascular development, OCT-based biomechanical imaging could be of particular interest. OCT elastography is an emerging functional approach to optically probe tissue elasticity [91]. Due to the high sensitivity to movements and the feasibility to incorporate different types of loading approaches, OCT-based elastography has found attractive applications in a number of biomedical fields [92], including embryonic heart biomechanics. Utilizing the heartbeat as natural loading, Li et al. demonstrated the capability of OCT in measuring the strain and strain rate in embryonic chick heart [93,94], which can be readily adopted for mouse embryonic heart wall analysis. Also, based on the pulse wave propagation, mechanical assessment can be conducted on the outflow tract of the chick embryonic heart, revealing the heart wall stiffness [95]. Similar analysis has been performed on the E9.5 mouse embryonic heart with an ultrafast OCT system [49]. As tissue stiffness, contractility, and blood flow shear stress have been implicated in direct regulation of cell differentiation, migration and gene expression during heart development, live mechanical analysis is of significant value for understanding early cardiogenesis. Therefore, we envision more technical development and application-driven studies in this area of quantitative biomechanical imaging in the future.

The use of computational modeling and numerical analysis [66] will further strengthen and elevate the spatiotemporal scale provided by OCT for cardiac biomechanical studies. Particularly, OCT has been combined with computational fluid dynamics to study the hemodynamics of the embryonic aortic arch [96]. Imaging-based computational analyses described the transitions of aortic arch patterning and hemodynamics in the early chick embryos at a number of developmental stages [97,98,99]. Such studies showed flow-induced wall shear stress as an important epigenetic factor in aortic arch regression and generation [97], revealed dynamics of shear stress at the critical stage of aortic arch morphogenesis [98], and identified hemodynamics as a functional biomarker for ethanol-induced abnormal aortic arch remodeling. These point to a clear path for integrating OCT and computational approaches to understand the role of hemodynamics in mammalian cardiogenesis with the mouse model.

Different imaging technologies have their unique advantages and limitations. Hybrid and multimodality strategy combining OCT with other methods has a potential to synergistically enhance the capabilities for cardiodynamic embryonic analysis. Liu et al. developed a dual modality system with OCT and photoacoustic tomography for imaging of the chick embryo in toto [100], acquiring complementary information for structural and functional analysis. Since photoacoustic imaging of the mouse embryo has been demonstrated [101,102], this multimodal approach should be applicable for mouse embryonic imaging as well. Recently, Brillouin microscopy has been combined with OCT to probe the elastic property of extracted mouse embryos, focusing on the neural tube [103,104]. With the further improvement in the imaging speed of Brillouin microscopy [105] as well as the integration of the two systems [106], OCT-guided direct biomechanical assessment in the beating mouse embryonic heart is potentially possible. In another multimodality configuration, the lack of molecular contrast from OCT was complemented by light-sheet microscopy as well as the establishment of rotation-based OCT imaging [107,108]. In addition to imaging, a number of manipulation methods have been established for mouse embryos, such as controlled microinjection to the blood circulation [109,110] and cardiac optogenetic pacing [111], which set a platform for mechanistic studies investigating the interplay between molecular and biomechanical factors in mammalian cardiogenesis.

As we consider the future, it is imminent that our understanding of cardiovascular gene regulation will improve and our methods to control genes to prevent congenital defects or improve cardiac performance will develop. Currently, the International Mouse Phenotyping Consortium is performing a tour de force to generate and phenotypically characterize 5000 knockout mouse lines and has already identified many involved in cardiovascular development [112]. OCT based cardiodynamic imaging is well positioned at this moment to handle the rapid requirement to functionally characterize cardiac phenotypes. OCT’s ability for multimodality will allow investigators to probe structural, functional, and molecular mechanisms that are being disrupted. The future of OCT cardiodynamic imaging is promising as more cardiovascular mouse biologists step into the multidisciplinary field of biophotonics to carry on and improve upon this work.
